# Índices Inflamatórios Sistêmicos como Novos Biomarcadores para Canal Arterial Hemodinamicamente Significativo

**DOI:** 10.36660/abc.20240211

**Published:** 2024-11-18

**Authors:** Ufuk Cakir, Cuneyt Tayman

**Affiliations:** 1 Ankara Bilkent City Hospital Division of Neonatology Department of Pediatrics Cankaya Ankara Turquia Ankara Bilkent City Hospital – Division of Neonatology, Department of Pediatrics, Cankaya, Ankara – Turquia

**Keywords:** Canal Arterial, Recém-Nascido, Recém-Nascido Prematuro, Inflamação

## Abstract

**Fundamento:**

Aumento da tensão de oxigênio e diminuição dos níveis de prostaglandina causam fechamento ductal. O papel diagnóstico dos índices inflamatórios sistêmicos no canal arterial hemodinamicamente significativo (hsPCA) em bebês prematuros é desconhecido.

**Objetivos:**

Nosso objetivo foi avaliar o papel dos índices inflamatórios sistêmicos na preditividade da hsPCA.

**Métodos:**

Bebês prematuros com semanas gestacionais (SG) <32 semanas foram avaliados retrospectivamente. Índices inflamatórios sistêmicos proporção neutrófilo-linfócito (NLR), proporção monócito-linfócito (MLR), proporção plaqueta-linfócito (PLR), índice de inflamação imune sistêmica (SII), valor de inflamação pan-imune (PIV) e índice de resposta à inflamação sistêmica (SIRI) foram calculados. Índices inflamatórios sistêmicos foram comparados entre os grupos hsPCA e não hsPCA. Um p < 0,05 foi considerado estatisticamente significativo.

**Resultados:**

Um total de 1228 pacientes foram incluídos no estudo, incluindo 447 pacientes no grupo hsPCA e 781 pacientes no grupo não-hsPCA. O valor PIV [mediana (Q1 - Q3): 5,18 (2,38-10,42)] no grupo hsPCA foi estatisticamente significativamente maior do que o valor PIV [mediana (Q1 - Q3): 3,52 (1,41-6,45)] no grupo não-hsPCA (p<0,001). De acordo com a análise ROC, o valor AUC do PIV para a previsibilidade de hsPCA foi de 0,618, e o nível de corte foi >8,66. Após até mesmo múltiplas análises de regressão logística, o PIV mostrou ser um parâmetro significativo para o diagnóstico de hsPCA (OR 1,972, IC 95% 1,114-3,011. p=0,001).

**Conclusões:**

Um alto valor de PIV pode ser um indicador de uso rápido, baixo custo, simples e facilmente acessível para o diagnóstico precoce de hsPCA.

## Graphical abstract

**Figura Central:**
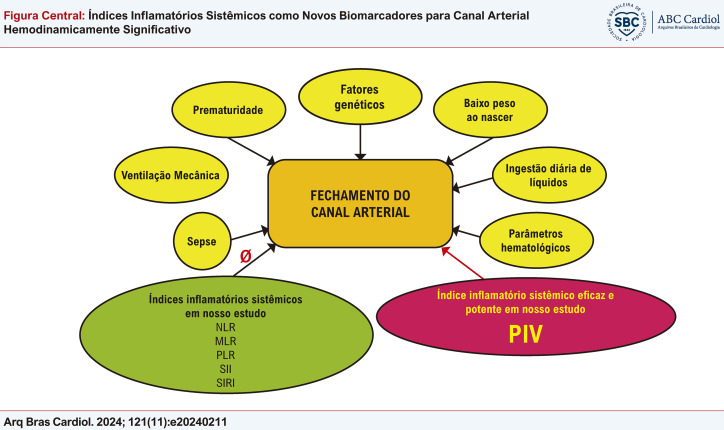
: Índices Inflamatórios Sistêmicos como Novos Biomarcadores para Canal Arterial Hemodinamicamente Significativo Resumo dos resultados do nosso estudo. MRL: razão monócitos/linfócitos; NRL: razão neutrófilos/linfócitos; PIV: valor de inflamação panimune; PLR: razão plaquetas/linfócitos; SII: Índice de inflamação imunológica sistêmica; SIRI: Índice de resposta à inflamação sistêmica.

## Introdução

O canal arterial (CA), que funciona abertamente na vida intrauterina, deve fechar após o nascimento. A falha no fechamento do CA na vida extrauterina causa um aumento no fluxo sanguíneo pulmonar e, consequentemente, uma diminuição no fluxo sanguíneo sistêmico. A instabilidade hemodinâmica devido ao canal arterial patente (PCA) está associada ao aumento de morbidades, como hemorragia pulmonar, disfunção renal, síndrome do desconforto respiratório (SDR), displasia broncopulmonar (DBP), hemorragia intraventricular (HIV), enterocolite necrosante (NEC), retinopatia da prematuridade (ROP), leucomalácia periventricular (PVL) e paralisia cerebral, além de maior mortalidade.^1^

A hipótese clássica no fechamento ductal é que a contração das células musculares lisas no CA é induzida por baixa tensão de oxigênio, diminuição da prostaglandina E2 (PGE2) e prostaciclina (PGI2). Essa contração causa baixa tensão de oxigênio local, desencadeando a morte celular e aumentando a liberação de fatores de crescimento induzíveis por baixa tensão de oxigênio. Então, ocorre o fechamento funcional do CA. Como resultado, ocorre a remodelação vascular e o fechamento anatômico do CA. Além da hipótese clássica de fechamento do CA, eletrólitos séricos, osmolaridade sérica, nível de hemoglobina, contagem de plaquetas e funções plaquetárias também podem afetar o fechamento do CA.^2-4^ No entanto, o efeito dos índices inflamatórios sistêmicos obtidos a partir de parâmetros de hemograma no fechamento do CA em bebês prematuros não é conhecido exatamente.

Recentemente, alguns estudos relacionados a alguns índices inflamatórios foram conduzidos no diagnóstico de infecção em adultos, avaliação da resposta ao tratamento da sepse e resultados clínicos, avaliação dos resultados clínicos da infecção por COVID-19, avaliação da fibrose pulmonar idiopática, hemorragia subaracnóidea, metástases tumorais, embolia pulmonar e mortalidade após cirurgia de revascularização do miocárdio. Para esse propósito, a razão neutrófilo-linfócito (NLR), razão monócito-linfócito (MLR) e razão plaqueta-linfócito (PLR) foram investigadas. Nos últimos anos, o uso de novos índices inflamatórios sistêmicos, como o índice de inflamação imune sistêmica (SII), o valor pan-imune-inflamação (PIV) e o índice de resposta à inflamação sistêmica (SIRI) atraiu atenção no diagnóstico de algumas doenças e na avaliação de resultados clínicos.^5-15^

No campo da neonatologia, alguns estudos relataram que altos valores de NLR podem estar associados à sepse de início precoce (SIP), taquipneia transitória do recém-nascido (TTN), ROP, HIV, NEC e resultados neonatais adversos.^16-20^ Além disso, um aumento no valor de PLR demonstrou estar associado a um aumento na frequência de restrição de crescimento intrauterino (RCIU), SIP e PCA.^20-24^ Sabe-se que a inflamação afeta o fechamento ductal. Índices inflamatórios sistêmicos são usados como um indicador da gravidade da inflamação. Os efeitos dos índices inflamatórios sistêmicos no fechamento ductal não são totalmente compreendidos. No entanto, não há estudos avaliando a relação entre índices inflamatórios sistêmicos como MLR, PIV, SII, SIRI e PCA hemodinamicamente significativa (hsPCA) em bebês prematuros. No presente estudo, objetivamos avaliar a relação entre seis índices inflamatórios sistêmicos e hsPCA em bebês prematuros <32 semanas gestacionais (SG).

## Métodos

### Desenho e plano de estudo

Nosso estudo foi realizado em bebês prematuros com SG <32 semanas em nossa unidade de terapia intensiva neonatal. Os dados dos pacientes foram obtidos retrospectivamente de registros hospitalares entre fevereiro de 2018 e outubro de 2021. Pacientes com anomalias congênitas importantes, cardiopatia congênita, SG ≥32 semanas e pacientes que morreram nos primeiros três dias após o parto sem diagnóstico de PCA foram excluídos do estudo. Características demográficas e desfechos clínicos, valores de hemograma completo (como dados de glóbulos brancos e plaquetas) de todos os pacientes incluídos no estudo foram registrados. Os pacientes incluídos no estudo foram tratados com os mesmos protocolos durante toda a hospitalização. Os pacientes inscritos foram divididos em dois grupos: hsPCA e não hsPCA. A aprovação ética foi obtida do comitê de ética local antes do estudo.

### Características demográficas e resultados clínicos

Dados de todos os pacientes, incluindo SG, peso ao nascer (PN), exposição pré-natal a esteroides, sexo, SDR, HIV (grau ≥ 3), NEC (grau > 2), DBP moderada/grave, ROP, SIP e sepse de início tardio (SIT) foram registrados.

### Morbidades prematuras

Pacientes que apresentavam dificuldade respiratória e necessitavam de tratamento com surfactante endotraqueal foram definidos como SDR.^25^ Pacientes com HIV grave (grau ≥ 3) por ultrassonografia craniana foram registrados.^26^ Bebês com NEC (grau ≥ 2), de acordo com achados clínicos e laboratoriais, foram registrados.^27^ Quando a idade pós-menstrual do prematuro atinge a 36ª semana, aqueles que necessitam de < 30% de oxigênio foram definidos como moderados, e aqueles que necessitaram de ≥30% de oxigênio ou suporte de pressão positiva foram definidos como DBP grave.^28^ Pacientes diagnosticados e tratados para ROP no exame de retina foram registrados.^29^ Sepse diagnosticada em <72 horas foi definida como SIP, e sepse reconhecida em ≥72 horas foi identificada como SIT.^30^

### Definição de ducto arterial persistente hemodinamicamente significativo

A ecocardiografia Doppler (ECHO) foi realizada para todos os prematuros nas primeiras 72 horas de vida pós-natal. Pacientes elegíveis foram diagnosticados com hsPCA de acordo com critérios clínicos e de ECHO. Pacientes que não preencheram os critérios para hsPCA no ECHO (por exemplo, diâmetro interno de PCA < 1,5 mm ou razão átrio esquerdo/raiz aórtica < 1,5 ou sem PCA) foram definidos como não-hsPCA (Tabela 1). Pacientes com PCA clinicamente e ecocardiograficamente significativa foram definidos como hsPCA. Esses pacientes primeiro receberam tratamento médico para fechamento ductal. Posteriormente, se a presença de hsPCA persistisse após dois cursos de tratamento médico, o tratamento de ligadura ductal era decidido e aplicado. A primeira opção no tratamento médico de PCA era o ibuprofeno. Se houvesse uma contraindicação para o uso de ibuprofeno, o paracetamol era usado como tratamento médico. As contraindicações do ibuprofeno foram definidas como sepse, sangramento ativo, trombocitopenia, coagulopatia, NEC, insuficiência renal e doença cardíaca congênita dependente do ducto.^31^

**Tabela 1:** – Canal arterial patente hemodinamicamente significativo

Características clínicas	Murmúrio
	Precórdio hiperdinâmico
	Pulsos pré-ductais delimitadores
	Piora do estado respiratório
	Pressão de pulso ampla
	Hipotensão
	Acidose metabólica
Características ecocardiográficas	Aumento da relação átrio esquerdo/raiz aórtica
	Cardiomegalia
	Shunting da esquerda para a direita
	Grande canal aberto (>1,5 mm)
	Reversão do fluxo nas principais artérias pós-ductais

### Análise de hemograma completo e índices inflamatórios sistêmicos

Amostras de sangue de todos os bebês prematuros nas primeiras 24 horas de vida foram coletadas em tubos de ácido etilenodiaminotetracético (EDTA) e foi realizada uma análise de hemograma completo.^20,21,32^Os valores de contagem de leucócitos (103 µ/L), contagem de neutrófilos (103 µ/L), contagem de monócitos (103 µ/L), contagem de linfócitos (103 µ/L), contagem de plaquetas (103 µ/L) e proporção de neutrófilos imaturos para total foram analisados com hemocitômetro automático Cell-Dyn 3700 (Abbott, Abbott Park, IL, EUA). NLR, fórmula NLR=N/L, fórmula PLR=P/L, fórmula MLR=M/L, fórmula SII=P x N/L, fórmula SIRI=N x M/L e PIV=P x N x M/L calculados usando as fórmulas. Como todos os índices inflamatórios são razões, eles não têm uma unidade.^33^ Os grupos hsPCA e não hsPCA foram comparados em termos de características demográficas e resultados clínicos, hemograma completo e índices inflamatórios sistêmicos.

### Análise estatística

Todos os dados foram analisados com o programa *Statistical Package for Social Sciences* (SPSS), versão 20.0 (SPSS Inc, Chicago, IL, EUA). A avaliação da conformidade das variáveis com a distribuição normal foi implementada com o teste visual (histograma) e o teste de Kolmogorov-Smirnov. O teste exato de Fisher ou o teste qui-quadrado de Pearson foi usado para a análise de variáveis categóricas. Um teste-t de Student não pareado ou o teste U de Mann-Whitney foi usado para a análise de variáveis contínuas. Variáveis contínuas com distribuição normal foram apresentadas como média ± desvio padrão (DP), variáveis com distribuição não normal foram destacadas como mediana e intervalo interquartil (IQR) (Q1 - Q3), e os resultados das variáveis categóricas foram apresentados como frequência. A regressão logística multivariada foi aplicada para identificar os fatores de risco independentes de hsPCA, como PN e SG. A análise das curvas *Receiver Operating Characteristics* (ROC) foi realizada para avaliar o nível de significância do parâmetro. A área sob a curva (AUC) e o intervalo de confiança (IC) de 95% da AUC, valores de corte, sensibilidade, especificidade, valor preditivo positivo (VPP) e valor preditivo negativo (VPN) foram calculados pela análise ROC. Um p < 0,05 foi considerado estatisticamente significativo.

## Resultados

**Figura 1:**
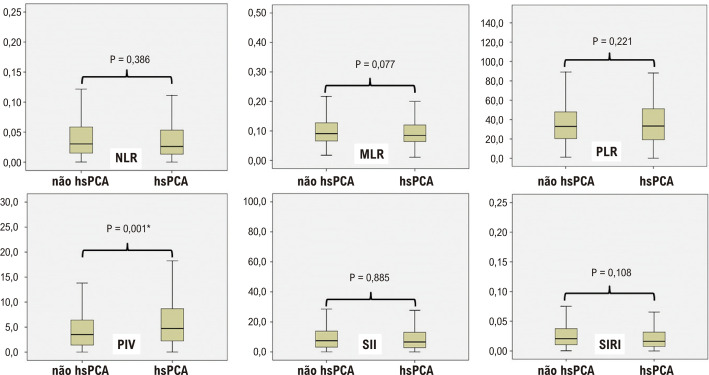
– Gráfico de caixa de índices inflamatórios sistêmicos com base na persistência do canal arterial hemodinamicamente significativa. *P<0,05 foi considerado estatisticamente significativo. hsPCA: persistência do canal arterial hemodinamicamente significativa; MLR: razão monócitos/linfócitos; NLR: razão neutrófilos/linfócitos; PIV: valor de inflamação panimune; PLR: razão plaquetas/linfócitos; SII: índice de inflamação imunológica sistêmica; SIRI: índice de resposta à inflamação sistêmica.

Durante o período do estudo, 1.379 bebês foram avaliados. De acordo com os critérios de exclusão, 151 recém-nascidos foram excluídos do estudo. Um total de 1.228 pacientes foram incluídos no estudo. Quatrocentos e quarenta e sete pacientes foram incluídos no grupo hsPCA e 781 pacientes no grupo não hsPCA. A frequência de hsPCA foi de 36,4% (447/1.228) em prematuros com SG < 32 semanas. O SG médio de todos os pacientes do estudo foi de 28,4 ± 1,3 semanas e o PN médio foi de 1.077 ± 234 g. Os resultados foram semelhantes entre os grupos em termos de SG, PN, esteroides pré-natais, gênero, NEC, SIP e SIT. A frequência de DBP, HIV, SDR e ROP no grupo hsPCA foi estatisticamente significativamente maior do que no grupo não hsPCA (Tabela 2). Contagens de leucócitos, plaquetas, neutrófilos, monócitos, linfócitos, razão de neutrófilos imaturos para total, NLR, MLR, PLR, SII e SIRI foram encontradas como semelhantes em ambos os grupos. O valor de PIV no grupo hsPCA foi estatisticamente significativamente maior do que o valor de PIV no grupo não-hsPCA (Tabela 3) (Figura 1 e Figura Central).

**Tabela 2:** – Variáveis demográficas e resultados clínicos

Características	não-hsPDA (n=781)	hsPDA (n=447)	Valor p
Semana gestacional, ^a^	28,5 ± 1,3	28,4 ± 1,4	0,066
Peso ao nascer, g ^a^	1082 ± 234	1068 ± 230	0,080
Esteroide pré-natal, n (%)	541 (69,2)	305 (68,2)	0,447
Gênero masculino, n (%)	412 (52,7)	219 (48,9)	0,205
DBP, n (%)	65(8,3)	119 (26,6)	<0,001*
HIV, n (%)	40 (5,1)	62 (13,8)	<0,001*
NEC, n (%)	14 (1,8)	10 (2.2)	0,711
SDR, n (%)	371 (47,5)	303 (67,7)	<0,001*
ROP, n (%)	44 (5,6)	57 (12,7)	<0,001*
SIP, n (%)	22 (2,8)	11 (2,4)	0,841
Tempo de permanência, n (%)	141 (18)	101 (22,5)	0,737

^a^média ± desvio padrão. *P<0,05 foi considerado estatisticamente significativo. DBP: displasia broncopulmonar; SIP: sepse de início precoce; HIV: hemorragia intraventricular; NEC: enterocolite necrosante; hsPCA: persistência do canal arterial hemodinamicamente significativa; SIT: sepse de início tardio; SDR: síndrome do desconforto respiratório; ROP: retinopatia da prematuridade.

**Tabela 3:** – Parâmetros leucocitários de acordo com persistência do canal arterial hemodinamicamente significativa

Parâmetros	não-hsPCA (n=781)	hsPCA (n=447)	Valor p
Contagem de leucócitos (10^3^ µ/L) ^a^	11h16 (7h21-16h00)	11h00 (8h20-16h01)	0,195
Contagem de plaquetas (10^3^ µ/L) ^a^	223 (82-301)	225 (99-309)	0,183
Contagem de neutrófilos (10^3^ µ/L) ^a^	0,22 (0,14-0,35)	0,23 (0,15-0,33)	0,566
Contagem de monócitos (10^3^ µ/L) ^a^	0,63 (0,43-0,99)	0,65 (0,42-1,01)	0,356
Contagem de linfócitos (10^3^ µ/L) ^a^	7,14 (5,25-10,8)	7,05 (4,71-10,63)	0,178
Proporção de neutrófilos imaturos para o total ^a^	0,05 (0,01-0,08)	0,06 (0,01-0,09)	0,322
NLR ^a^	0,03 (0,01-0,06)	0,02 (0,01-0,04)	0,386
MLR ^a^	0,09 (0,06-0,13)	0,08 (0,06-0,12)	0,077
PLR ^a^	33,47 (20,37-48,41)	33,52 (19,21-51,51)	0,221
PIV ^a^	3,52 (1,41-6,45)	5,18 (2,38-10,42)	<0,001*
SII ^a^	7,63 (3,25-13,98)	6,59 (2,81-13,24)	0,885
SIRI ^a^	0,02 (0,01-0,03)	0,02 (0,01-0,03)	0,108

^a^ mediana (intervalo interquartil). *P < 0,05 foi considerado estatisticamente significativo. hsPCA: persistência do canal arterial hemodinamicamente significativa; MLR: razão monócitos/linfócitos; NLR: razão neutrófilos/linfócitos; PIV: valor de inflamação panimune; PLR: razão plaquetas/linfócitos; SII: índice de inflamação imunológica sistêmica; SIRI: índice de resposta à inflamação sistêmica.

Quatrocentos e seis pacientes foram incluídos no grupo hsPCA com tratamento médico e 41 pacientes no grupo hsPCA com ligadura cirúrgica. O dia médio de ligadura cirúrgica foi de 22±11 dias. Não houve diferença estatisticamente significativa entre os dois grupos em termos de índices inflamatórios sistêmicos. Os resultados são apresentados na Tabela 4.

**Tabela 4:** – Parâmetros leucocitários de acordo com o tratamento médico e ligadura cirúrgica em canal arterial patente hemodinamicamente significativo

Parâmetros	hsPCA com tratamento médico (n=406)	hsPCA com ligadura cirúrgica (n=41)	Valor p
Contagem de leucócitos (103 µ/L) ^a^	11h00 (8h00-16h00)	12h90 (8h60-20h30)	0,375
Contagem de plaquetas (103 µ/L) ^a^	220 (95-277)	246 (104-299)	0,291
Contagem de neutrófilos (103 µ/L) ^a^	0,22 (0,15-0,33)	0,30 (0,19-0,44)	0,068
Contagem de monócitos (103 µ/L) ^a^	0,61 (0,42-1,01)	0,72 (0,47-1,14)	0,281
Contagem de linfócitos (103 µ/L) ^a^	6,98 (4,71-10,62)	9,69 (5,12-12,80)	0,314
Proporção de neutrófilos imaturos para o total ^a^	0,03 (0,01-0,04)	0,03 (0,01-0,04)	0,401
NLR ^a^	0,02 (0,01-0,04)	0,03 (0,01-0,05)	0,961
MLR ^a^	0,08 (0,04-0,12)	0,09 (0,04-0,13)	0,162
PLR ^a^	35,12 (20,37-48,41)	27,42 (19,21-41,97)	0,708
PIV ^a^	4,95 (1,41-9,48)	6,08 (3,32-15,78)	0,349
SII ^a^	7,63 (10,72)	6,47 (3,66-12,89)	0,730
SIRI ^a^	0,01 (0,01-0,03)	0,02 (0,01-0,07)	0,274

*^a^ mediana (intervalo interquartil). hsPCA: persistência do canal arterial hemodinamicamente significativa; MLR: razão monócitos/linfócitos; NLR: razão neutrófilos/linfócitos; PIV: valor de inflamação panimune; PLR: razão plaquetas/linfócitos; SII: índice de inflamação imunológica sistêmica; SIRI: índice de resposta à inflamação sistêmica.*

**Figura 2:**
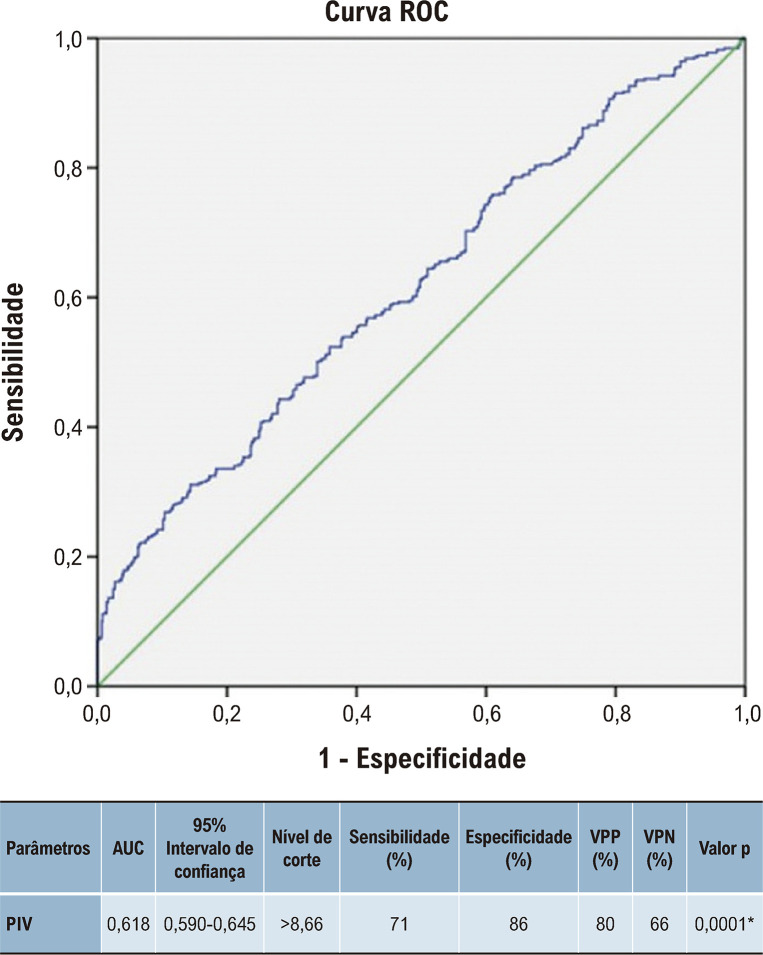
– Curvas características de operação do receptor para valor de inflamação panimune em canal arterial patente hemodinamicamente significativo em < 32 semanas gestacionais. AUC: área sob a curva; VPP: valor preditivo positivo; VPN: valor preditivo negativo; PIV:valor de inflamação pan-imune.

A análise ROC foi realizada para PIV para avaliar a preditividade de hsPCA em bebês <32 SG. AUC, intervalo de confiança de 95%, sensibilidade, especificidade, VPP, valores de VPN e gráfico ROC de PIV são apresentados na Figura 2. Após múltiplas análises de regressão logística, o PIV mostrou-se um parâmetro significativo para o diagnóstico de hsPCA (OR 1,972, IC 95% 1,114-3,011. p=0,001).

## Discussão

Em nosso estudo, o valor preditivo e diagnóstico dos índices inflamatórios sistêmicos em hsPCA foi avaliado. De acordo com os resultados da literatura, nosso estudo foi o primeiro a avaliar o efeito de seis índices inflamatórios sistêmicos em prematuros com hsPCA. Com base em nossos resultados, foi descoberto que NLR, MLR, PLR, SII e SIRI não puderam ser usados como um indicador significativo para a predição de hsPCA. Descobrimos que o PIV foi o parâmetro efetivo e único para a predição de hsPCA nos seis índices inflamatórios sistêmicos.

Foi afirmado que altos valores de NLR, MLR e PLR podem ter papéis importantes na determinação do prognóstico de doenças como sepse, doenças cardiovasculares e malignidades em adultos.^7,8,10^No entanto, no campo da neonatologia, há poucos dados sobre a relação entre NLR, MLR, PLR e doenças neonatais. Altos valores de NLR são mostrados como associados à sepse neonatal, TTN, ROP, HIV e NEC.^17-20,34,35^ Pelo contrário, estudos estão relatando que NLR, MLR e PLR não são significativos para o diagnóstico de SIP e ROP.^11,23^ Além disso, NLR não foi considerado um parâmetro importante em recém-nascidos com PCA em 28-37 semanas de gestação.^32^ Estudos recentes em bebês prematuros relataram que altos valores de SII podem ser novos marcadores para o diagnóstico de SDR, e altos valores de SIRI podem ser um novo marcador para a predição e diagnóstico de DBP moderada a grave.^36,37^

Devido à inconsistência nos resultados de estudos que avaliam NLR, MLR e PLR tanto em neonatologia quanto em outras faixas etárias, a busca por novos índices inflamatórios sistêmicos que possam ser usados no diagnóstico e prognóstico de doenças continua. Nesse contexto, foi determinado que SII alto pode ser um parâmetro eficaz na determinação do prognóstico e mortalidade em pacientes com câncer, pacientes com embolia pulmonar e esclerose múltipla.^11,12,38^ Foi determinado que um aumento no SIRI pode ser um indicador diagnóstico precoce para infecção da corrente sanguínea em pacientes que requerem hemodiálise e um marcador prognóstico em pacientes com doenças cardiovasculares e câncer.^13,14,39^ Por outro lado, PIV é um parâmetro mais novo, e foi relatado que seu alto nível pode estar associado ao prognóstico de pacientes com câncer.^11,40^

O número de estudos avaliando seis índices inflamatórios sistêmicos como um fator diagnóstico e prognóstico em algumas doenças é muito limitado. Urbanowicz et al. relataram que seis índices inflamatórios sistêmicos estavam aumentados em pacientes submetidos a bypass da artéria coronária no período pós-operatório, e NLR e SIRI poderiam ser marcadores eficazes para sobrevivência pós-operatória.^13^ Zinellu et al. descobriram que pacientes com fibrose pulmonar idiopática com um PIV (também chamado de índice agregado de inflamação sistêmica (AISI)) ≥434 tiveram um tempo de sobrevivência mediano mais curto. NLR, MLR, PLR, SII e SIRI não foram associados à sobrevivência em pacientes com fibrose pulmonar idiopática também.^15^ Ceran et al. determinaram que os valores de NLR, SII, SIRI e PIV foram maiores em pacientes com encefalopatia hipóxico-isquêmica neonatal (HIE) em comparação ao grupo controle, mas o PLR foi menor do que o grupo controle. Após hipotermia terapêutica, um aumento significativo foi encontrado no valor de PLR, juntamente com uma diminuição considerável nos valores de NLR, SII, SIRI e PIV. Além disso, foi relatado que o valor de NLR é o parâmetro significativo entre seis índices inflamatórios sistêmicos, que tem o maior poder preditivo para HIE.^33^ Descobrimos que apenas o valor de PIV foi o índice inflamatório sistêmico efetivo no diagnóstico de hsPCA.

Na hipóxia-isquemia cerebral, há uma expressão aumentada de citocinas inflamatórias e uma resposta inflamatória à lesão envolvendo neutrófilos e linfócitos. Portanto, o parâmetro NLR pode se tornar um preditor mais forte em pacientes com HIE.^33^ Assim como na fisiopatologia da HIE, a hipóxia é um fator na fisiopatologia do fechamento ductal. Considerando o aumento de neutrófilos, monócitos e a diminuição da contagem de linfócitos devido à baixa tensão de oxigênio, os índices inflamatórios sistêmicos podem se tornar um marcador para o diagnóstico de hsPCA.^33^ Enquanto mediadores pró-inflamatórios e baixa tensão de oxigênio afetam o fechamento ductal, o efeito das células leucocitárias no fechamento ductal não é totalmente conhecido. A esse respeito, não é fácil interpretar os índices inflamatórios sistêmicos obtidos da contagem de leucócitos em doenças com fisiopatologia complexa, como PCA. Nesse caso, pode ser mais preciso encontrar o parâmetro com o maior valor preditivo para hsPCA.

Proliferação acelerada de megacariócitos, aumento da destruição plaquetária e trombocitose estão associados à inflamação e isquemia. Como o aumento ou diminuição da contagem de plaquetas e a diminuição da contagem de linfócitos estão associados à agregação e à inflamação, eles podem ser usados como marcadores de fator de risco em hsPCA. Portanto, índices inflamatórios sistêmicos (PLR, SII e PIV) nos quais as plaquetas estão incluídas podem ser usados no diagnóstico de hsPCA. As plaquetas desempenham um papel importante tanto estrutural quanto funcionalmente no fechamento do CA.^2^ Em relação a isso, foi determinado que o PLR foi maior no grupo hsPCA no 1º, 2º, 3º e 7º dias após o nascimento em bebês com SG <34 semanas. Semelhante aos nossos resultados, a contagem de plaquetas não foi associada ao hsPCA.^21^ No entanto, em nosso estudo, o valor do PLR não foi considerado um parâmetro significativo no diagnóstico de hsPCA. Isso pode ocorrer porque nossos pacientes são mais imaturos e as amostras de sangue são coletadas apenas nas primeiras 24 horas.

A elevação do SII foi considerada um parâmetro significativo na mortalidade associada à cirurgia de revascularização do miocárdio em adultos.^13^ Foi relatado que o valor do SII é um marcador valioso no diagnóstico precoce de sepse neonatal em recém-nascidos a termo com cardiopatia congênita.^35^ No entanto, a relação entre o valor do SII e a PCA não foi avaliada antes. Em nossos resultados, foi descoberto que o parâmetro do SII não foi valioso na predição de hsPCA. Em alguns estudos com adultos, o PIV demonstrou ser o valor prognóstico efetivo entre seis índices inflamatórios em pacientes com câncer colorretal metastático e fibrose pulmonar idiopática.^11,15^ Em nosso estudo, o valor do PIV, calculado pela inclusão de todos os parâmetros leucocitários, foi considerado efetivo na predição de hsPCA. De acordo com nossos resultados, baixa tensão de oxigênio e inflamação podem resultar em aumento da apoptose de linfócitos, contagens de neutrófilos e monócitos também.^33^ Em nossos dados, apenas a relação entre índices inflamatórios sistêmicos e hsPCA foi avaliada. Entre esses seis índices, o PIV se destaca como o único parâmetro associado à hsPCA. O PIV pode ter sido um parâmetro significativo em relação tanto à PCA quanto à baixa tensão de oxigênio e inflamação, com possivelmente todos os linfócitos, neutrófilos, monócitos e plaquetas sendo afetados. Essa hipótese deve ser avaliada em futuros estudos prospectivos.

Consequentemente, acredita-se que tanto as células leucocitárias responsáveis pela inflamação quanto as plaquetas desempenham um papel no processo de fechamento ductal.^2,41^ Assim, o valor do PIV pode surgir como um parâmetro importante na previsibilidade do hsPCA. Como em nossos resultados, embora não haja diferença nas contagens de neutrófilos, monócitos, linfócitos e plaquetas entre os grupos, o PIV pode ser o parâmetro efetivo para hsPCA quando todos os parâmetros são avaliados juntos. No entanto, nossos resultados são válidos apenas para índices inflamatórios sistêmicos obtidos nas primeiras 24 horas em prematuros com SG <32 semanas e hsPCA. Durante 24 horas após o parto prematuro, os índices inflamatórios podem aumentar devido à SIP, por exemplo, da corioamnionite, que é frequentemente uma causa de prematuridade. Seria mais adequado analisar a proporção de neutrófilos totais imaturos, que é conhecida como um indicador precoce de SIP.^42^ Em nossos resultados, tanto a proporção de neutrófilos imaturos quanto a proporção de SIP foram semelhantes entre os grupos, mas o valor de PIV foi encontrado como um parâmetro que pode ser usado no acompanhamento de hsPCA.

Outro resultado em nosso estudo foi que o valor de corte diagnóstico do PIV de hsPCA foi >8,66, que foi menor do que os valores de corte em outros estudos envolvendo >410 para HIE, >390 para câncer colorretal metastático e >663 para mortalidade em pacientes com cirurgia de revascularização do miocárdio.^11,13,33^ A principal razão para essa diferença nos valores de corte para PIV nos estudos pode ser devido à idade dos pacientes, diferenças no diagnóstico, tempo de coleta de sangue e ser a termo ou prematuro. Como as contagens de neutrófilos são baixas e as contagens de monócitos e linfócitos são altas em bebês a termo em valores de intervalo de referência normais em comparação com adultos.^43^ Além disso, o número de monócitos e linfócitos diminui fisiologicamente nos dias pós-natais após o nascimento.^23^ À medida que a SG diminui em bebês prematuros, os valores normais de referência de monócitos e linfócitos aumentam enquanto os valores de neutrófilos diminuem. Além disso, há uma ligeira diminuição nos valores normais de referência das contagens de plaquetas em bebês prematuros em comparação com bebês a termo.^44^ De acordo com esses dados, ao interpretar os índices inflamatórios sistêmicos obtidos do hemograma em recém-nascidos, a SG e o tempo de coleta de sangue devem ser levados em consideração em doenças com fisiopatologia complexa, como a CA.

No presente estudo, objetivamos identificar índices inflamatórios sistêmicos que pudessem predizer a presença de hsPCA. Além disso, avaliamos a relação entre índices inflamatórios sistêmicos e hsPCA recebeu tratamento médico, e hsPCA necessitou de ligadura cirúrgica. O índice inflamatório sistêmico foi semelhante entre os dois grupos. Em nosso estudo, os índices inflamatórios sistêmicos foram avaliados apenas nas primeiras 24 horas de vida. Como hsPCA foi diagnosticado em torno de 3 dias, o índice inflamatório sistêmico pode ter um valor preditivo no diagnóstico de hsPCA. A ligadura cirúrgica foi aplicada após dois cursos de tratamento médico; assim, o dia da ligadura foi estendido até 3 semanas pós-natais. Não foi um parâmetro eficaz para avaliar a necessidade de ligadura cirúrgica, que foi a morbidade tardia associada a hsPCA. Sugerimos que pacientes com hsPCA podem ser investigados para decidir sobre ligadura ductal durante o tratamento médico pós-natal avaliando os valores de PIV.

À medida que o SG diminui, a frequência de hsPCA aumenta. No entanto, não há um único parâmetro com alto poder de predição para ajudar a determinar qual bebê prematuro terá hsPCA. Esse parâmetro pode auxiliar o clínico a estimar o paciente em risco de hsPCA durante o acompanhamento do bebê prematuro. Esse tipo de parâmetro também pode lançar luz sobre as morbidades da prematuridade que podem estar associadas à hsPCA.^45^ Nesse sentido, o PIV pode ser um novo parâmetro para hsPCA. Além do importante valor de contribuição do PIV na predição de hsPCA, o PIV é um parâmetro barato e de rápido acesso que não requer custo adicional. De acordo com nossos resultados, embora o PIV tenha sido um parâmetro eficaz na predição de hsPCA, não foi um parâmetro útil que indica a necessidade de ligadura ductal. No entanto, o valor do PIV avaliado no primeiro dia pode prever hsPCA, que foi uma morbidade precoce de bebês prematuros. Não foi um parâmetro eficaz para avaliar a necessidade de ligadura cirúrgica, que foi a morbidade tardia associada à hsPCA. Sugerimos que pacientes com hsPCA podem ser investigados para decidir sobre ligadura ductal durante o tratamento médico pós-natal avaliando os valores de PIV.

Nosso estudo teve algumas limitações, pois continha dados de um único centro e foi projetado retrospectivamente. Amostras de sangue foram coletadas de todos os bebês prematuros nas primeiras 24 horas de vida. Assim, o efeito dos índices inflamatórios sistêmicos no CA em dias pós-natais subsequentes não pôde ser avaliado. Além disso, os dados sobre o índice inflamatório sistêmico antes e depois do tratamento não puderam ser analisados.

## Conclusões

Nosso estudo é o primeiro a avaliar seis índices inflamatórios sistêmicos no diagnóstico e predição de hsPCA em bebês prematuros. Entre seis índices inflamatórios sistêmicos, o PIV foi considerado o parâmetro efetivo no diagnóstico e predição de hsPCA. Portanto, o PIV pode ser facilmente acessível, usado rapidamente e um indicador simples e de baixo custo para o diagnóstico de hsPCA. No entanto, o papel do PIV como um marcador preditivo e diagnóstico para hsPCA precisa ser confirmado por outros estudos prospectivos.
