# Supplementary material for: Índices Inflamatórios Sistêmicos como Novos Biomarcadores para Canal Arterial Hemodinamicamente Significativo
Source: Arq Bras Cardiol. 2024 Nov 18;121(11):e20240211. [Article in Portuguese] doi: 10.36660/abc.20240211 (PMC11634317; doi:10.36660/abc.20240211)
Supplement: Translation

# Translation: Systemic Inflammatory Indices as New Biomarkers for Hemodynamically Significant Ductus Arteriosus

**DOI:** 10.36660/abc.20240211i

**Authors:** Ufuk Cakir, Cuneyt Tayman

**Affiliations:** 1 Turkey Ankara Bilkent City Hospital – Division of Neonatology, Department of Pediatrics, Cankaya, Ankara – Turkey

**Keywords:** Ductus Arteriosus, Newborn Infant, Premature Infant, Inflammation

## Abstract

**Background:**

Increased oxygen tension and decreased prostaglandin levels cause ductal closure. The diagnostic role of systemic inflammatory indices in hemodynamically significant ductus arteriosus (hsPDA) in premature infants is unknown.

**Objectives:**

We aimed to evaluate the role of systemic inflammatory indices in the predictivity of hsPDA.

**Methods:**

Premature infants with gestational weeks (GW) of <32 weeks were evaluated retrospectively. Systemic inflammatory indices neutrophil-lymphocyte ratio (NLR), monocyte-lymphocyte ratio (MLR), platelet-lymphocyte ratio (PLR), systemic immune-inflammation index (SII), pan-immune- inflammation value (PIV), and systemic inflammation response index (SIRI) were calculated. Systemic inflammatory indices were compared between hsPDA and non-hsPDA groups. A p <0.05 was considered as statistically significant.

**Results:**

A total of 1228 patients were included in the study, including 447 patients in the hsPDA group and 781 patients in the non-hsPDA group. The PIV value [median (Q1 - Q3): 5.18 (2.38-10.42)] in the hsPDA group was statistically significantly higher than the PIV value [median (Q1 - Q3): 3.52 (1.41-6.45)] in the non-hsPDA group (p<0.001). According to the ROC analysis, the AUC value of PIV for the predictivity of hsPDA was 0.618, and the cutoff level was >8.66. After even multiple logistic regression analyses, PIV was shown to be a significant parameter for the diagnosis of hsPDA (OR 1.972, 95% CI 1.114-3.011. p=0.001).

**Conclusions:**

A high PIV value may be a quickly used indicator with low-cost, simple, and easily accessible for the early diagnosis of hsPDA.

## Introduction

The ductus arteriosus (DA), which functions openly in intrauterine life, should close after birth. Failure of the closure of DA in extrauterine life causes an increase in pulmonary blood flow and, consequently, a decrease in systemic blood flow. Hemodynamic instability due to patent ductus arteriosus (PDA) is associated with increased morbidities such as pulmonary hemorrhage, kidney dysfunction, respiratory distress syndrome (RDS), bronchopulmonary dysplasia (BPD), intraventricular hemorrhage (IVH), necrotizing enterocolitis (NEC), retinopathy of prematurity (ROP), periventricular leukomalacia (PVL) and cerebral palsy in addition to higher mortality.^1^

The classical hypothesis in ductal closure is that the contraction of smooth muscle cells in the DA is induced by low oxygen tension, decreased prostaglandin E2 (PGE2), and prostacyclin (PGI2). This contraction causes local low oxygen tension, triggering cell death and increasing the release of low oxygen tension -inducible growth factors. Then, the functional closure of the DA takes place. As a result, vascular remodeling and anatomical closure of the DA occur. Apart from the classical DA closure hypothesis, serum electrolytes, serum osmolality, hemoglobin level, platelet count, and platelet functions may also affect DA closure.^2-4^ However, the effect of systemic inflammatory indices obtained from hemogram parameters on DA closure in preterm infants is not known exactly.

Recently, some studies related to some inflammatory indices have been conducted in the diagnosis of infection in adults, evaluation of sepsis treatment response and clinical results, evaluation of clinical results of COVID-19 infection, evaluation of idiopathic pulmonary fibrosis, subarachnoid hemorrhage, tumor metastases, pulmonary embolism and mortality after coronary artery bypass surgery. For this purpose, neutrophil-lymphocyte ratio (NLR), monocyte-lymphocyte ratio (MLR), and platelet-lymphocyte ratio (PLR) were investigated. In recent years, the use of new systemic inflammatory indices such as the systemic immune-inflammation index (SII), pan-immune-inflammation value (PIV), and systemic inflammation response index (SIRI) has attracted attention in the diagnosis of some diseases and the evaluation of clinical outcomes.^5-15^

In the field of neonatology, some studies have reported that high NLR value may be associated with early onset sepsis (EOS), transient tachypnea of the newborn (TTN), ROP, IVH, NEC, and adverse neonatal outcomes.^16-20^ Moreover, an increase in PLR value has been shown to be associated with an increase in the frequency of intrauterine growth restriction (IUGR), EOS, and PDA.^20-24^ It is known that inflammation affects ductal closure. Systemic inflammatory indices are used as an indicator of the severity of inflammation. The effects of systemic inflammatory indices on ductal closure are not fully understood. However, there are no studies evaluating the relationship between systemic inflammatory indices such as MLR, PIV, SII, SIRI, and hemodynamically significant PDA (hsPDA) in premature infants. In the present study, we aimed to evaluate the relationship between six systemic inflammatory indices and hsPDA in premature babies <32 gestational weeks (GW).

## Methods

### Study design and plan

Our study was carried out in premature babies with a GW of <32 weeks in our neonatal intensive care unit. The data of the patients were obtained retrospectively from hospital records between February 2018 and October 2021. Patients with major congenital anomalies, congenital heart disease, GW ≥32 weeks, and patients who died within the first three days after delivery without a diagnosis of PDA were excluded from the study. Demographic characteristics and clinical outcomes, complete blood count values (such as white blood cell and platelet data) of all patients included in the study were recorded. Patients included in the study were treated with the same protocols throughout hospitalization. The enrolled patients were divided into two groups: hsPDA and non-hsPDA. Ethical approval was obtained from the local ethics committee before the study.

### Demographical features and clinical outcomes

Data for all patients, including GW, birth weight (BW), antenatal steroid exposure, gender, RDS, IVH (≥3 grade), NEC (grade >2), moderate/severe BPD, ROP, EOS, and late-onset sepsis (LOS) were recorded.

### Premature morbidities

Patients who had respiratory distress and required endotracheal surfactant treatment were defined as RDS.^25^ Patients with severe IVH (≥3 grade) by cranial ultrasonography were recorded.^26^ Babies with NEC (≥2 grade), according to clinical and laboratory findings, were registered.^27^ When the postmenstrual age of the premature infant reaches the 36^th^ week, those who need <30% oxygen were defined as moderate, and those requiring ≥30% oxygen or positive pressure support were defined as severe BPD.^28^ Patients diagnosed and treated for ROP in the retinal examination were recorded.^29^ Sepsis diagnosed in <72 hours was defined as EOS, and sepsis recognized at ≥72 hours was identified as LOS.^30^

### Definition of hemodynamically significant patent ductus arteriosus

Doppler echocardiography (ECHO) was performed for all premature infants in the first 72 hours of postnatal life. Eligible patients were diagnosed with hsPDA according to clinical and ECHO criteria. Patients who did not meet the criteria for hsPDA in ECHO (e.g., PDA internal diameter <1.5 mm or left atrium/aortic root ratio <1.5 or without PDA) were defined as non-hsPDA (Table 1). Patients with both clinically and echocardiographically significant PDA were defined as hsPDA. These patients first received medical treatment for ductal closure. Subsequently, if the presence of hsPDA persisted after two courses of medical treatment, ductal ligation treatment was decided and applied. The first option in the medical treatment of PDA was ibuprofen. If there was a contraindication for ibuprofen use, paracetamol was used as medical treatment. Ibuprofen contraindications were defined as sepsis, active bleeding, thrombocytopenia, coagulopathy, NEC, renal failure, and duct-dependent congenital heart disease.^31^

**Table 1:** – Hemodynamically significant patent ductus arteriosus

Clinical features	Murmur
	Hyperdynamic precordium
	Bounding pre-ductal pulses
	Worsening respiratory status
	Wide pulse pressure
	Hypotension
	Metabolic acidosis
Echocardiographic features	Increased left atrium to aortic root ratio
	Cardiomegaly
	Left-to-right shunting
	Large open ductus (>1.5 mm)
	Reversal of flow in post-ductal major arteries

### Complete blood count analysis and systemic inflammatory indices

Blood samples from all premature babies within the first 24 hours of life were taken into ethylenediaminetetraacetic acid (EDTA) tubes, and a complete blood count analysis was performed.^20,21,32^ Leukocyte count (103 µ/L), neutrophil count (103 µ/L), monocyte count (103 µ/L), lymphocyte count (103 µ/L), platelet count (103 µ/L) values, and immature to total neutrophil ratio were analyzed with Cell-Dyn 3700 automatic hemocytometer (Abbott, Abbott Park, IL, USA). NLR, NLR=N/L formula, PLR=P/L formula, MLR=M/L formula, SII=P x N/L formula, SIRI=N x M/L formula, and PIV=P x N x M/L calculated using the formulas. Since all inflammatory indices are ratios, they do not have a unit.^33^ The hsPDA and non-hsPDA groups were compared in terms of demographical features and clinical outcomes, complete blood count, and systemic inflammatory indices.

### Statistical analysis

All data were analyzed with the Statistical Package for Social Sciences (SPSS), version 20.0 (SPSS Inc, Chicago, IL, USA) program. Evaluation of the conformity of the variables to the normal distribution was implemented with both visual (histogram) and Kolmogorov-Smirnov test. Fisher’s Exact test or Pearson Chi-Square test was used for the analysis of categorical variables. An unpaired Student´s t-test or Mann-Whitney U test was used for the analysis of continuous variables. Normally distributed continuous variables were presented as mean ± standard deviation (SD), non-normally distributed variables were highlighted as the median and interquartile range (IQR) (Q1 - Q3), and the results of categorical variables were shown as frequency. Multivariate logistic regression was applied to identify the independent risk factors of hsPDA, like as BW and GW. Receiver operating characteristics (ROC) curves analysis was performed to evaluate the significance level of the parameter. The area under the curve (AUC) and the 95% confidence interval (CI) of the AUC, cutoff values, sensitivity, specificity, positive predictive value (PPV), and negative predictive value (NPV) were calculated by ROC analysis. A p <0.05 was considered statistically significant.

## Results

During the study period, 1379 infants were evaluated. According to the exclusion criteria, 151 newborn infants were excluded from the study. A total of 1228 patients were enrolled in the study. Four hundred forty-seven patients were included in the hsPDA group, and 781 patients in the non-hsPDA group. The frequency of hsPDA was found to be 36.4% (447/1228) in preterms with a GW of <32 weeks. The mean GW of all study patients was 28.4±1.3 weeks, and the mean BW was 1077±234 g. Results were similar between the groups in terms of GW, BW, antenatal steroid, gender, NEC, EOS, and LOS. The frequency of BPD, IVH, RDS, and ROP in the hsPDA group was found to be statistically significantly higher than in the non-hsPDA group (Table 2). Leukocyte, platelet, neutrophil, monocyte, lymphocyte counts, immature to total neutrophil ratio, NLR, MLR, PLR, SII, and SIRI were found to be similar in both groups. The PIV value in the hsPDA group was statistically significantly higher than the PIV value in the non-hsPDA group (Table 3) (Figure 1 and Central Illustration).

**Table 2:** – Demographic variables and clinical outcomes

Characteristics	non-hsPDA (n=781)	hsPDA (n=447)	p value
Gestational week, ^a^	28.5 ± 1.3	28.4 ± 1.4	0.066
Birth weight, g ^a^	1082 ± 234	1068 ± 230	0.080
Antenatal steroid, n (%)	541 (69.2)	305 (68.2)	0.447
Male gender, n (%)	412 (52.7)	219 (48.9)	0.205
BPD, n (%)	65(8.3)	119 (26.6)	<0.001*
IVH, n (%)	40 (5.1)	62 (13.8)	<0.001*
NEC, n (%)	14 (1.8)	10 (2.2)	0.711
RDS, n (%)	371 (47.5)	303 (67.7)	<0.001*
ROP, n (%)	44 (5.6)	57 (12.7)	<0.001*
EOS, n (%)	22 (2.8)	11 (2.4)	0.841
LOS, n (%)	141 (18)	101 (22.5)	0.737

^a^ mean ± standard deviation. *P<0.05 was considered statically significant. BPD: bronchopulmonary dysplasia; EOS: early onset sepsis; IVH: intraventricular hemorrhage; NEC: necrotising enterocolitis; hsPDA: hemodynamically significant patent ductus arteriosus; LOS: late onset sepsis; RDS: respiratory distress syndrome; ROP: retinopathy of prematurity.

**Table 3:** – Leukocyte parameters according to hemodynamically significant patent ductus arteriosus

Parameters	non-hsPDA (n=781)	hsPDA (n=447)	p value
Leukocyte count (10^3^ µ/L) ^a^	11h16 (7h21-16h00)	11h00 (8h20-16h01)	0.195
Platelet count (10^3^ µ/L) ^a^	223 (82-301)	225 (99-309)	0.183
Neutrophil count (10^3^ µ/L) ^a^	0.22 (0.14-0.35)	0.23 (0.15-0.33)	0.566
Monocyte count (10^3^ µ/L) ^a^	0.63 (0.43-0.99)	0.65 (0.42-1.01)	0.356
Lymphocyte count (10^3^ µ/L) ^a^	7.14 (5.25-10.8)	7.05 (4.71-10.63)	0.178
Immature to total neutrophil ratio ^a^	0.05 (0.01-0.08)	0.06 (0.01-0.09)	0.322
NLR ^a^	0.03 (0.01-0.06)	0.02 (0.01-0.04)	0.386
MLR ^a^	0.09 (0.06-0.13)	0.08 (0.06-0.12)	0.077
PLR ^a^	33.47 (20.37-48.41)	33.52 (19.21-51.51)	0.221
PIV ^a^	3.52 (1.41-6.45)	5.18 (2.38-10.42)	<0.001*
SII ^a^	7.63 (3.25-13.98)	6.59 (2.81-13.24)	0.885
SIRI ^a^	0.02 (0.01-0.03)	0.02 (0.01-0.03)	0.108

^a^ median (interquartile range). *P <0.05 was considered statically significant. hsPDA: hemodynamically significant patent ductus arteriosus; MLR: monocyte to lymphocyte ratio; NLR: neutrophil to lymphocyte ratio; PIV: pan immune inflammation value; PLR: platelet to lymphocyte ratio; SII: systemic immune inflammation index; SIRI: systemic inflammation response index.

**Figure 1:** – Box plot of systemic inflammatory indices based on hemodynamically significant patent ductus arteriosus. *P <0.05 was considered statically significant. hsPDA: hemodynamically significant patent ductus arteriosus; MLR: monocyte to lymphocyte ratio; NLR: neutrophil to lymphocyte ratio; PIV: pan immune inflammation value; PLR: platelet to lymphocyte ratio; SII: systemic immune inflammation index; SIRI: systemic inflammation response index.

Four hundred and six patients were included in the hsPDA with medical treatment group and 41 patients in the hsPDA with surgical ligation group. The mean surgical ligation day was 22±11 days. There was no statistically significant difference between the two groups in terms of systemic inflammatory indices. The results are given in Table 4.

**Table 4:** – Leukocyte parameters according to medical treatment and surgical ligation in hemodynamically significant patent ductus arteriosus

Parameters	hsPDA with medical treatment (n=406)	hsPDA with surgical ligation (n=41)	p value
Leukocyte count (10^3^ µ/L) ^a^	11h00 (8h00-16h00)	12h90 (8h60-20h30)	0.375
Platelet count (10^3^ µ/L) ^a^	220 (95-277)	246 (104-299)	0.291
Neutrophil count (10^3^ µ/L) ^a^	0.22 (0.15-0.33)	0.30 (0.19-0.44)	0.068
Monocyte count (10^3^ µ/L) ^a^	0.61 (0.42-1.01)	0.72 (0.47-1.14)	0.281
Lymphocyte count (10^3^ µ/L) ^a^	6.98 (4.71-10.62)	9.69 (5.12-12.80)	0.314
Immature to total neutrophil ratio ^a^	0.03 (0.01-0.04)	0.03 (0.01-0.04)	0.401
NLR ^a^	0.02 (0.01-0.04)	0.03 (0.01-0.05)	0.961
MLR ^a^	0.08 (0.04-0.12)	0.09 (0.04-0.13)	0.162
PLR ^a^	35.12 (20.37-48.41)	27.42 (19.21-41.97)	0.708
PIV ^a^	4.95 (1.41-9.48)	6.08 (3.32-15.78)	0.349
SII ^a^	7.63 (10.72)	6.47 (3.66-12.89)	0.730
SIRI ^a^	0.01 (0.01-0.03)	0.02 (0.01-0.07)	0.274

*^a^ median (interquartile range). hsPDA: hemodynamically significant patent ductus arteriosus; MLR: monocyte to lymphocyte ratio; NLR: neutrophil to lymphocyte ratio; PIV: pan immune inflammation value; PLR: platelet to lymphocyte ratio; SII: systemic immune inflammation index; SIRI: systemic inflammation response index.*

ROC analysis was performed for PIV to evaluate the predictivity of hsPDA in infants <32 GW. AUC, 95% confidence interval, sensitivity, specificity, PPV, NPV values, and ROC graph of PIV are presented in Figure 2. After even multiple logistic regression analyses, PIV was shown to be a significant parameter for the diagnosis of hsPDA (OR 1.972, 95% CI 1.114-3.011. p=0.001).

**Figure 2:** – Receiver operating characteristic curves for pan immune inflammation value in hemodynamically significant patent ductus arteriosus in < 32 gestational weeks. AUC: area under curve; PPV: positive predictive value; NPV: negative predictive value; PIV: pan immune inflammation value.

## Discussion

In our study, the predictive and diagnostic value of systemic inflammatory indices in hsPDA was evaluated. According to literature results, our study was the first to assess the effect of six systemic inflammatory indices in preterms with hsPDA. Based on our results, it was found that NLR, MLR, PLR, SII, and SIRI could not be used as a meaningful indicator for the prediction of hsPDA. We found that the PIV was the effective and only parameter for the prediction of hsPDA in the six systemic inflammatory indices.

It has been stated that high NLR, MLR, and PLR values may have important roles in determining the prognosis of diseases such as sepsis, cardiovascular diseases and malignancies in adults.^7,8,10^ However, in the field of neonatology, there is little data on the relationship between NLR, MLR, PLR, and neonatal diseases. High NLR values are shown to be associated with neonatal sepsis, TTN, ROP, IVH, and NEC.^17-20,34,35^ On the contrary, studies are reporting that NLR, MLR, and PLR are not significant for the diagnosis of EOS and ROP.^11,23^ Additionally, NLR was not found to be an important parameter in newborns with PDA at 28-37 weeks of gestation.^32^Recent studies in preterm infants have reported that high SII values may be new markers for the diagnosis of RDS, and high SIRI values may be a new marker for the prediction and diagnosis of moderate to severe BPD.^36,37^

Due to the inconsistency in the results of studies evaluating NLR, MLR, and PLR in both neonatology and other age groups, the search for new systemic inflammatory indices that can be used in the diagnosis and prognosis of diseases continues. In this context, it has been determined that high SII may be an effective parameter in determining the prognosis and mortality in patients with cancer, patients with pulmonary embolism, and multiple sclerosis.^11,12,38^ It has been determined that an increase in SIRI may be an early diagnostic indicator for bloodstream infection in patients requiring hemodialysis and a prognostic marker in patients with cardiovascular diseases and cancer.^13,14,39^ On the other hand, PIV is a newer parameter, and it has been reported that its high level may be associated with the prognosis of patients with cancer.^11,40^

The number of studies evaluating six systemic inflammatory indices as a diagnostic and prognostic factor in some diseases is very limited. Urbanowicz et al. reported that six systemic inflammatory indices were increased in patients who underwent coronary artery bypass in the postoperative period, and NLR and SIRI could be effective markers for postoperative survival.^13^ Zinellu et al. found that patients with idiopathic pulmonary fibrosis with a PIV (also called the aggregate index of systemic inflammation (AISI)) ≥434 had a shorter median survival time. NLR, MLR, PLR, SII, and SIRI were not associated with survival in patients with idiopathic pulmonary fibrosis as well.^15^ Ceran et al. determined that NLR, SII, SIRI, and PIV values were higher in patients with neonatal hypoxic-ischemic encephalopathy (HIE) compared to the control group, but PLR was lower than the control group. After therapeutic hypothermia, a significant increase was found in the PLR value, along with a considerable decrease in NLR, SII, SIRI, and PIV values. Moreover, it has been reported that the NLR value is the significant parameter among six systemic inflammatory indices, which has the highest predictive power for HIE.^33^ We found that only the PIV value was the effective systemic inflammatory indices in the diagnosis of hsPDA.

In cerebral hypoxia-ischemia, there is an increased expression of inflammatory cytokines and an inflammatory response to injury involving neutrophils and lymphocytes. Therefore, the NLR parameter may become a stronger predictor in patients with HIE.^33^ As in the pathophysiology of HIE, hypoxia is a factor in the pathophysiology of ductal closure. Considering the increased neutrophil, monocytes, and decreased lymphocyte counts due to low oxygen tension, systemic inflammatory indices may become a marker for the diagnosis of hsPDA.^33^ While proinflammatory mediators and low oxygen tension affect ductal closure, the effect of leukocyte cells on ductal closure is not fully known. In this respect, it is not easy to interpret the systemic inflammatory indices obtained from the leukocyte count in diseases with complex pathophysiology, such as PDA. In this case, it may be more accurate to find the parameter with the highest predictive value for hsPDA.

Accelerated megakaryocyte proliferation, increased platelet destruction, and thrombocytosis are associated with inflammation and ischemia. Since increased or decreased platelet count and decreased lymphocyte count are associated with both aggregation and inflammation, they can be used as risk factor markers in hsPDA. Therefore, systemic inflammatory indices (PLR, SII, and PIV) in which platelets are included may be used in the diagnosis of hsPDA. Platelets play an important role both structurally and functionally in DA closure.^2^ In relation to that, it was determined that PLR was higher in the hsPDA group on the 1^st^, 2^nd^, 3^rd,^ and 7^th^ days after birth in babies with a GW of <34 weeks. Similar to our results, platelet count was not found to be associated with hsPDA.^21^ However, in our study, PLR value was not found to be a significant parameter in the diagnosis of hsPDA. This may be due to the fact that our patients are more immature, and blood samples are taken only within the first 24 hours.

SII elevation was found to be a significant parameter in mortality associated with coronary artery bypass surgery in adults.^13^ It has been reported that SII value is a valuable marker in the early diagnosis of neonatal sepsis in term newborns with congenital heart disease.^35^ However, the relationship between SII value and PDA has not been evaluated before. In our results, it was found that the SII parameter was not valuable in the prediction of hsPDA. In some adult studies, PIV was shown to be the effective prognostic value among six inflammatory indices in patients with metastatic colorectal cancer and idiopathic pulmonary fibrosis.^11,15^ In our study, the PIV value, calculated by including all leukocyte parameters, was found to be effective in predicting hsPDA. According to our results, low oxygen tension and inflammation may result in increased lymphocyte apoptosis neutrophil and monocyte counts as well.^33^ In our data, only the relationship between systemic inflammatory indices and hsPDA was evaluated. Among these six indices, PIV stands out as the only parameter associated with hsPDA. PIV may have been a significant parameter in relation to both PDA and low oxygen tension and inflammation, with possibly all lymphocytes, neutrophils, monocytes, and platelets being affected. This hypothesis should be evaluated in future prospective studies.

Consequently, both leukocyte cells responsible for inflammation and platelets are thought to play a role in the ductal closure process.^2,41^ Thus, the PIV value may come forward as an important parameter in hsPDA predictivity. As in our results, although there is no difference in neutrophil, monocytes, lymphocyte, and platelet counts between the groups, PIV may be the effective parameter for hsPDA when all parameters are evaluated together. However, our results are only valid for systemic inflammatory indices obtained within the first 24 hours in premature infants with GW <32 weeks and hsPDA. During 24 hours after premature birth, inflammatory indices could rise due to EOS, for example, from chorioamnionitis, which is often a cause of prematurity. It would be further to analyze the immature total neutrophil ratio, which is known as an early indicator of EOS.^42^ In our results, both immature to total neutrophil ratio and EOS ratio were similar between the groups, but the PIV value was found as a parameter that can be used in hsPDA follow-up.

Another result in our study was that the diagnostic PIV cutoff value of hsPDA was >8.66, which was lower than the cutoff values in other studies involving >410 for HIE, >390 for metastatic colorectal cancer, and >663 for mortality in patients with coronary artery bypass surgery.^11,13^ The main reason for this difference in cutoff values for PIV in the studies may be due to the age of the patients, differences in diagnosis, time of blood collection, and being term or preterm. As neutrophil counts are low and monocyte and lymphocyte counts are high in term infants at normal reference range values compared to adults.^43^ Moreover, the number of monocytes and lymphocytes decreases physiologically in the postnatal days after birth.^23^ As the GW decreases in preterm babies, the normal reference values of monocytes and lymphocytes increase while neutrophil values decrease. In addition, there is a slight decrease in the normal reference values of platelet counts in preterm babies compared to term babies.^44^ According to these data, when interpreting the systemic inflammatory indices obtained from the blood count in newborns, the GW and blood collection time must be taken into account in diseases with complex pathophysiology, such as DA.

In the present study, we aimed to identify systemic inflammatory indices that could predict the presence of hsPDA. Moreover, we evaluated the relationship between systemic inflammatory indices and hsPDA received medical treatment, and hsPDA required surgical ligation. Systemic inflammatory index were similar between both groups. In our study, systemic inflammatory indices were evaluated only within the first 24 hours of life. As hsPDA was diagnosed around 3 days, the systemic inflammatory index may have a predictive value in the diagnosis of hsPDA. Surgical ligation was applied after two courses of medical treatment; thus, the ligation day was extended up to postnatal 3 weeks. It was not an effective parameter to evaluate the need for surgical ligation, which was the late morbidity associated with hsPDA. We suggest that patients with hsPDA may be investigated for deciding ductal ligation during postnatal medical treatment by evaluating PIV values.

As the GW decreases, the frequency of hsPDA increases. However, there is no single parameter with a high power of predictivity to help determine which premature baby will have hsPDA. Such a parameter may assist the clinician in estimating the patient at risk for hsPDA during the follow-up of the preterm infant. Such a kind of this parameter may also shed light on the morbidities of prematurity that may be associated with hsPDA.^45^ In this regard, PIV may be a novel parameter for hsPDA. Besides the important contributing value of PIV in the predictivity of hsPDA, PIV is a cheap, quickly accessible parameter that does not require additional cost. According to our results, although PIV was an effective parameter in the predictivity of hsPDA, it was not a useful parameter indicating the need for ductal ligation. However, the PIV value evaluated within the first day can predict hsPDA, which was an early morbidity of preterm infants. It was not an effective parameter to assess the need for surgical ligation, which was the late morbidity associated with hsPDA. We suggest that patients with hsPDA may be investigated for deciding ductal ligation during postnatal medical treatment by evaluating PIV values.

Our study had some limitations, as it contained data from a single center and was designed retrospectively. Blood samples were taken from all premature infants within the first 24 hours of life. Thus, the effect of systemic inflammatory indices on DA in subsequent postnatal days could not be evaluated. In addition, data on systemic inflammatory index before and after treatment could not be analyzed.

## Conclusions

Our study is the first to evaluate six systemic inflammatory indices in the diagnosis and prediction of hsPDA in premature infants. Among six systemic inflammatory indices, PIV was found to be the effective parameter in the diagnosis and prediction of hsPDA. Therefore, PIV can be easily accessible, quickly used, and a low-cost, simple indicator for the diagnosis of hsPDA. However, the role of PIV as a predictive and diagnostic marker for hsPDA needs to be confirmed by other prospective studies.
